
JOURNAL INFORMATION
==============================
NLM Title Abbreviation: Food Sci Nutr
Journal ID: Food Sci Nutr
NLM Journal ID: 101605473
Journal ID: fsn3

Food Science & Nutrition

EISSN: 2048-7177
Publisher: Wiley

ARTICLE INFORMATION
==============================
PMCID: PMC4048603
DOI: 10.1002/fsn3.61
Article version: 1
Subjects: Issue Information

Issue Information

Print publication date: 2014 May
Electronic publication date: 2014 May 13
Volume: 2
Issue: 3
First page: i
Last page: iii
Copyright: © 2014 Food Science & Nutrition published by Wiley Periodicals, Inc.
Copyright year: 2014
License: This is an open access article under the terms of the Creative Commons Attribution License, which permits use, distribution and reproduction in any medium, provided the original work is properly cited.
License URL: https://creativecommons.org/licenses/by/3.0/

==============================
Volume 2, Number 3

### Food Science & Nutrition

Open Access

Editor-in-Chief
Dr. Y. Martin Lo

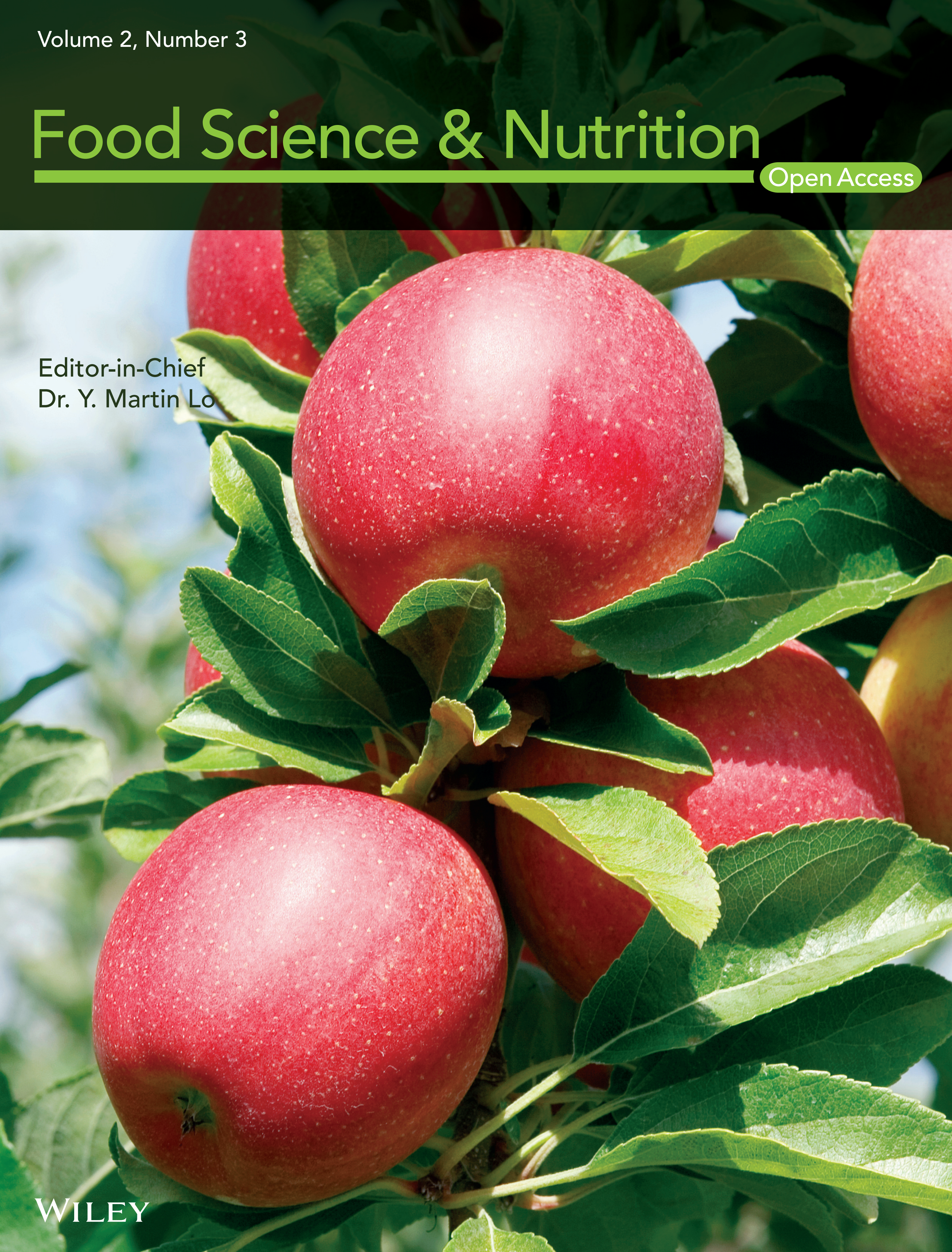

WILEY

### Food Science & Nutrition

Open Access

### Aims and Scope

*Food Science & Nutrition* is a peer-reviewed journal for rapid dissemination of research in all areas of food science and nutrition. The journal considers submissions of quality papers describing the results of fundamental and applied research related to all aspects of human food and nutrition, as well as interdisciplinary research that spans these two fields. Topics include, but are not limited to, the following areas:

- Chemistry of food and its biochemical interactions
- Food microbiology, safety, and risk assessment
- Metabolic, molecular, and genetic mechanisms in nutrition
- Safety and security analysis of global food supplies
- Food preservation, storage, and hurdle technology
- Food toxicology
- Engineering of food processing technologies
- Handling and packaging of foods
- Quality assurance of food products
- Biotechnology as it relates to food production and processing
- Food oral processing, rheology, and other texture-related studies
- Health and nutritional implications of food, functional foods, nutraceuticals, and supplements
- Bioavailability and disease prevention
- Nutritional methodologies, behaviors, and modeling
- Sensory and consumer science
- Community and international nutrition
- Enology and fermentation technology
- Food and dietary supplement ingredient regulatory science
- Health claims
- Agriculture research on plant production, utilization, biomass, and environment
- Commentaries on controversial issues in food science and nutrition
- Interdisciplinary research spanning food science and nutrition

*Food Science & Nutrition* publishes original research articles, systematic reviews, meta-analyses, and research methods papers, along with invited editorials and commentaries. Original research papers must report well-conducted research with conclusions supported by the data presented in the paper.

*Food Science & Nutrition* is a Wiley Open Access journal, one of a new series of peer-reviewed titles publishing quality research with speed and efficiency. For further information visit the Wiley Open Access website at http://www.wileyopenaccess.com.

### Open Access and Copyright

All articles published by *Food Science & Nutrition* are fully open access: immediately freely available to read, download, and share. All *Food Science & Nutrition* articles are published under the terms of the Creative Commons Attribution License which permits use, distribution, and reproduction in any medium, provided the original work is properly cited.

Copyright on any research article in a journal published by *Food Science & Nutrition* is retained by the author(s). Authors grant Wiley a license to publish the article and identify itself as the original publisher. Authors also grant any third party the right to use the article freely as long as its integrity is maintained and its original authors, citation details and publisher are identified. Further information about open access license and copyright can be found at http://www.wileyopenaccess.com/details/content/12f25db4c87/Copyright–License.html.

### Purchasing Print Reprints

Print reprints of Wiley Open Access articles can be purchased from corporatesales@wiley.com.

### Disclaimer

The Publisher and Editors cannot be held responsible for errors or any consequences arising from the use of information contained in this journal; the views and opinions expressed do not necessarily reflect those of the Publisher and Editors, neither does the publication of advertisements constitute any endorsements by the Publisher and Editors of the products advertised.

Wiley Open Access articles posted to repositories or websites are without warranty from Wiley of any kind, either express or implied, including, but not limited to, warranties of merchantability, fitness for a particular purpose, or non-infringement. To the fullest extent permitted by law Wiley disclaims all liability for any loss or damage arising out of, or in connection with, the use of or inability to use the content.

**Editor-in-Chief**
Y. Martin Lo
*Address correspondence to the Editorial Office: foodsci@wiley.com*

**Associate Editor**
Bernadene Magnuson
*University of Toronto, Canada*

**Editorial Board**

Yi Fang Chu
*Pepsi Co Nutrition, USA*
Hong Fu
*Fuzhou University, China*
Carmen Gomes
*Texas A & M, USA*
Dallas Hoover
*University of Delaware, USA*
Scott Jordan
*Health Canada, Canada*
Marilena Marino
*Università degli Studi di Udine, Italy*
Jennifer McEntire
*Leavitt Partners Center for Food Safety, USA*
Alexandra Oliveira
*University of Alaska, USA*
Stephan Rössner
*Karolinska Institutet, Sweden*
Sanem Soysal
*Yedipete University, Turkey*
Fidel Toldrá
*Instituto de Agroquímica y Tecnología de Alimentos (CSIC), Spain*
Kinga Topolska
*University of Agriculture in Krakow, Poland*
Thomas Wang
*United States Department of Agriculture, USA*
Youfa Wang
*Johns Hopkins University, USA*
Baojun (Bruce) Xu
*Beijing Normal University-Hong Kong Baptist University United International College, China*
Qixin Zhong
*University of Tennessee, USA*
Kequan (Kevin) Zhou
*Wayne State University, USA*
